# Novel Chromogens for Immunohistochemistry in Spatial Biology

**DOI:** 10.3390/cells13110936

**Published:** 2024-05-29

**Authors:** Bipin Gupta, George Yang, Marc Key

**Affiliations:** 1 Diagnostic BioSystems, Pleasanton, CA 94588, USA; george.yang@dbiosys.com; 2 Key Biomedical Services, Ojai, CA 93023, USA; key@keybiomedical.com

**Keywords:** multiplex, immunohistochemistry, chromogens, brightfield, spatial biology

## Abstract

Spatial relations between tumor cells and host-infiltrating cells are increasingly important in both basic science and clinical research. In this study, we have tested the feasibility of using standard methods of immunohistochemistry (IHC) in a multiplex staining system using a newly developed set of chromogenic substrates for the peroxidase and alkaline phosphatase enzymes. Using this approach, we have developed a set of chromogens characterized by (1) providing fine cellular detail, (2) non-overlapping spectral profiles, (3) an absence of interactions between chromogens, (4) stability when stored, and (5) compatibility with current standard immunohistochemistry practices. When viewed microscopically under brightfield illumination, the chromogens yielded the following colors: red, black, blue, yellow, brown, and green. By selecting compatible color combinations, we have shown feasibility for four-color multiplex staining. Depending on the particular type of analysis being performed, visual analysis, without the aid of computer-assisted image analysis, was sufficient to differentiate up to four different markers.

## 1. Introduction

There is a substantial body of literature describing the intra-tumor heterogeneity of both tumor and host cells as well as their interactions. Extensive efforts have been made to analyze the various cell types, locations, and associations within the tumor architecture. Such methods have focused primarily on single-cell measurements by flow cytometry or nucleic acid sequencing [1,2]. However, such methods based on dissociated cells fail to take into account the spatial relationships between the various cell types.

Discovering the molecular mechanisms affecting cell growth and development is critical in understanding disease processes, and this understanding can ultimately lead to better treatments. For example, in tumors there is an intimate relationship between the tumor cells and their surroundings. This can be understood to reflect a critical connection between the tumor cells and their environment. For example, some types of immunotherapy rely upon the treatment’s ability to interfere with certain interactions between tumor cells and immune cells [3]. It is now apparent that the characterization of the tumor microenvironment and the cell populations residing therein is critical to our understanding of these diseases.

Spatial immunohistochemistry (IHC) has now become a useful tool for analyzing morphological patterns [4,5] and cellular microenvironment [6,7]. In particular, methods utilizing immunofluorescence have proven extremely valuable in identifying and quantifying host-infiltrating immune cells [8,9]. One of the advantages of utilizing fluorochromes in this context is their narrow bandwidth, enabling multiple labels to be simultaneously visualized within a single tissue. However, despite these advantages, light microscopy still remains the method of choice for most pathologists as the morphological information provided by this method is critical for a primary diagnosis. Pathologists have been evaluating cells in tissue sections using classical antibody- and morphology-based staining methods for nearly 100 years. In clinical practice, these evaluations were frequently performed using chromogenic IHC methods that allowed for the assessment of one or two proteins within a single tissue slide. While conventional IHC methods using chromogenic labels can capture spatial organization, these methods are limited by the availability of robust chromogens that can be combined together within a single stain to produce multiplex staining images.

Multispectral imaging has promise for overcoming some of these limitations. Multispectral imaging can be used with both fluorescence and brightfield microscopy methods, although fewer chromogens are available for brightfield applications, and their absorbance spectra are relatively broad compared to most fluorochromes. Recent studies were able to overcome some of these limitations by the use of a new family of chromogens with narrow spectral absorption and appropriately matched brightfield illumination channels [10]. Further developments of brightfield multispectral imaging may allow the integration of this important technology into general pathology [11,12]. However, at present an objective, measurable, and reproducible means of evaluating spatial heterogeneity at the single-cell level by brightfield IHC remains a challenging goal.

## 2. Materials and Methods

### 2.1. Sample Preparation

All tissue specimens were obtained from surgical samples as paraffin blocks. Tissues had been formalin-fixed and paraffin-embedded (FFPE) using standard histological methods. Tissue sections were prepared at 4 µ, adhered to positively charged microscope slides, and stored at room temperature until the time of staining. At the time of staining, tissue sections were deparaffinized through graded solutions of xylene and alcohol and then rehydrated in deionized water. After deparaffinization, in order to firmly attach the tissues to the microscope slide during the staining procedure, the slides and the tissues were treated with an adherence-promoting reagent (Tissue Glue, Diagnostic BioSystems, Pleasanton, CA USA) according to the manufacturer’s instructions. Deparaffinized tissue sections were then subjected to antigen retrieval by submerging slides in a solution of Tris-EDTA, pH 9.0, and heating in a pressure cooker at 15 psi for 15 min. Slides in the antigen-retrieval solution were allowed to cool at room temperature until the pressure was relieved. Slides were then placed into a Tris-buffered wash solution and cooled at room temperature for five minutes.

### 2.2. Antibodies

Antibodies were screened from tonsils as single stains to determine optimal staining concentrations. All antibodies were obtained from Diagnostic BioSystems, Pleasanton, CA, USA, and are listed in Table 1.

### 2.3. Reagents

Table 2 lists reagents used. All reagents were obtained from Diagnostic BioSystems.

### 2.4. Chromogens

Table 3 lists all chromogens used. All chromogens were obtained from Diagnostic BioSystems.

### 2.5. Immunohistochemistry

Sequential multiplex staining was used because of its flexibility for using any antibody or enzyme system. The following steps were employed using a manual method at room temperature:
StepName1Primary Antibody2Rinse3Polymer (enzyme-conjugated secondary antibody)4Rinse5Substrate/Chromogen6Rinse7Elution Buffer

### 2.6. Peroxidase Method

Incubation time for primary antibodies and polymers was 20 min. Incubation time for substrate/chromogens for horseradish peroxidase (HRP) was 5 min.

### 2.7. Alkaline Phosphatase Method

Incubation time for primary antibodies and polymers was 20 min. Incubation time for substrate/chromogens for alkaline phosphatase (AP) polymers was 10 min.

### 2.8. Elution Buffer

The elution buffer was composed of 0.25% sodium dodecyl sulfate (SDS) in 0.1 M glycine at pH 2.0 [13]. Previous experiments had shown that an incubation time of 10 min at 50 °C was sufficient to remove all primary antibodies and secondary polymers from the stained slides.

### 2.9. Multiplex Staining

After a single sequence of staining and elution, slides were then re-stained with a different primary antibody using the same sequence as previously described but instead using a different colored chromogen. This sequence was repeated multiple times until up to four different chromogens had been applied to the tissues, each chromogen detecting a different antigen based on the specificity of the primary antibody.

### 2.10. Microscopic Evaluation

Slides were mounted using an ImmunoHisto-Sealer (Diagnostic BioSystems, Part No. K076) followed by a permanent mounting medium, according to the manufacturer’s instructions. Mounted slides were viewed using brightfield microscopy at 100× and 400× magnification. Slides were evaluated for specific staining, background staining, and chromogen mixing as indicated by the mixed colors. Specific staining was graded on a scale of 0 to 3 in 0.5 grade increments, with 0 indicating no staining, 1 indicating light staining, 2 indicating moderate staining, and 3 indicating strong staining.

## 3. Results

**Figure 1:**
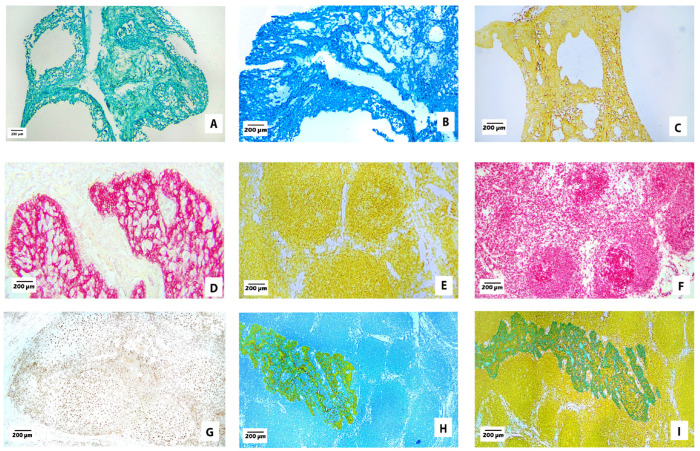
Optimization of chromogens on tonsil tissue. Tonsil tissue stained with chromogens (100×). (**A**) Pan-cytokeratin (AE1/AE3) with green-HRP, (**B**) pan-cytokeratin (AE1/AE3) with blue-HRP, (**C**) pan-cytokeratin (AE1/AE3) with yellow-HRP, (**D**) pan-cytokeratin (AE1/AE3) with red-AP, (**E**) B-cell (CD20) with yellow-HRP, (**F**) B-cell (CD20) with red-AP, (**G**) macrophage (CD163) with DAB, (**H**) pan-cytokeratin (AE1/AE3) with yellow-HRP and B-cell (CD20) with blue-HRP, and (**I**) pan-cytokeratin (AE1/AE3) with green-HRP and B-cell (CD20) with yellow-HRP.

Antibodies were tested on tonsil tissue as single stains and double stains to determine the optimal concentrations and to evaluate each chromogen for sensitivity, background, and fine cellular detail. Representative images for some of the chromogens are shown in Figure 1. Immunohistochemical staining with pan-cytokeratin on tonsil tissue is shown in Figure 1A–D, depicting chromogens for green-HRP, blue-HRP, yellow-HRP, and red-AP, respectively. These figures show the expected specific staining of surface and crypt epithelium with minimal background staining of non-epithelial elements. Therefore, these chromogens can be used to identify cells of epithelial origin, including most carcinomas. Figure 1E,F show staining results for B-lymphocytes in tonsil tissue with chromogens for yellow-HRP and red-AP. A typical staining pattern shows an abundance of B-lymphocytes in germinal centers and mantle zones of lymphoid follicles, with a lesser distribution of positive cells within the interfollicular spaces. Figure 1H,I show a combination of pan-cytokeratin and B-lymphocyte markers. In Figure 1H, pan-cytokeratin is stained with yellow-HRP and B-lymphocytes are stained with blue-HRP. In Figure 1I, pan-cytokeratin is stained with green-HRP and B-lymphocytes are stained with yellow-HRP.

Next, chromogens were evaluated for their ability to withstand the elution buffer without fading. The single stains were incubated in elution buffer for 10 min at 50 °C. Photographic images were compared both before and after treatment with the elution buffer. Certain chromogens showed a partial loss of signal after incubation with the elution buffer as shown below in Table 4.

Because certain chromogens showed a partial loss of staining in the elution buffer, these chromogens were either omitted or used as final stains in a multi-stain sequence. Chromogens showing a partial loss of signal included PermaRed-HRP, PermaGreen-HRP, and PermaBlack-HRP.

**Figure 2:**
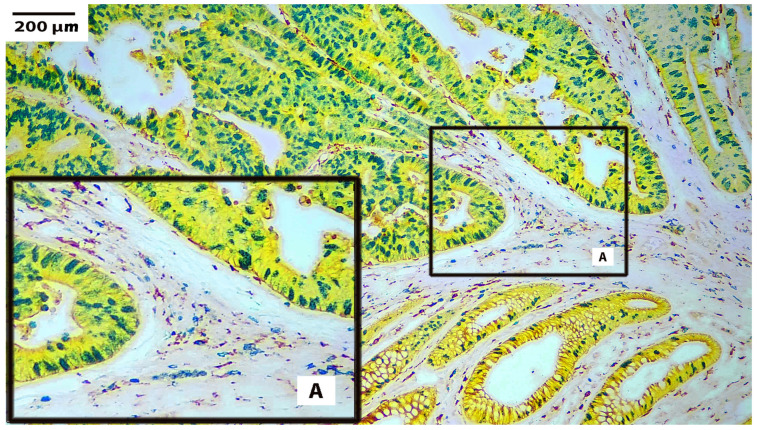
Four-plex stain on colorectal carcinoma using chromogens for HRP. Colorectal carcinoma (100×). Pan-cytokeratin (AE1/AE3) stained with yellow-HRP. Macrophage (CD163) stained with DAB. B-cell (CD20) stained with blue-HRP. Proliferation marker (Ki67) stained with green-HRP. Box A shows an area of the tumor at its original location (smaller Box A) and a higher magnification of the selected area (larger Box A).

**Figure 3:**
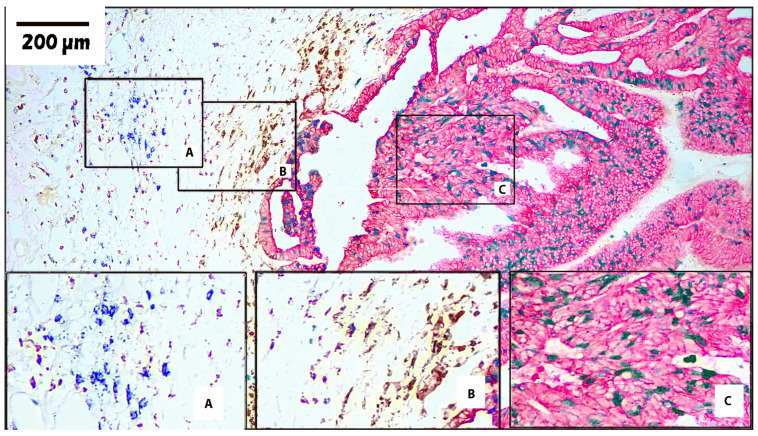
Four-plex stain on colorectal carcinoma using chromogens for AP and HRP. Colorectal carcinoma (100×). Pan-cytokeratin (AE1/AE3) stained with red-AP. Macrophage (CD163) stained with DAB. B-cell (CD20) stained with blue-HRP. Proliferation marker (Ki67) stained with green-HRP. Box A shows an area of predominantly B-cells (CD20). Box B shows an area of predominantly macrophages (CD163). Box C shows an area of predominantly tumor cells (AE1/AE3) that are also positive for the proliferation marker (Ki67). Small boxes A, B, and C show the tumor areas in their original locations, and larger boxes A, B, and C show a higher magnification of the same corresponding areas.

**Figure 4:**
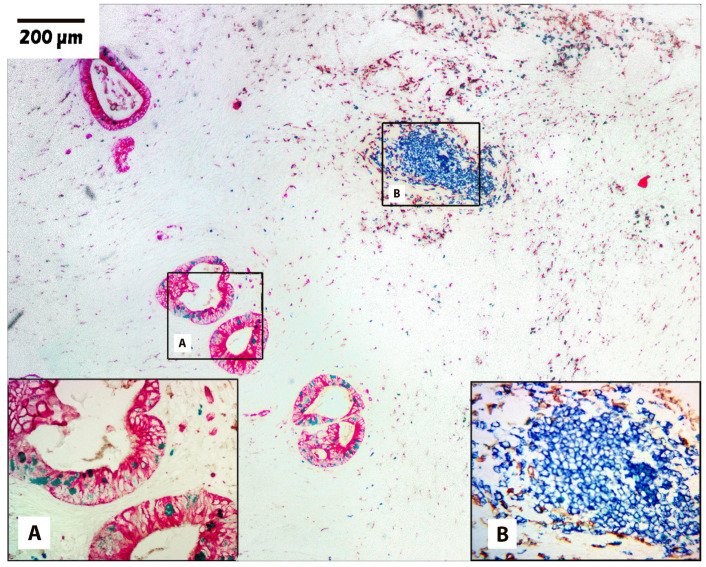
Four-plex stain of colorectal carcinoma showing inflammatory cells. Colorectal carcinoma (100×) showing an area of tumor stroma with an extensive inflammatory cell infiltrate. Tumor cells are stained with red-AP for pan-cytokeratin (AE1/AE3) and green-HRP for the proliferation marker (Ki67). Macrophages (CD68) are stained with DAB and B-cells (CD79a) are stained with blue-HRP. The small boxes A and B show the tumor areas at their original locations and the large boxes A and B show the same corresponding areas at a higher magnification.

Using the information gathered from these initial experiments, the optimal panels were constructed. Table 5 shows representative panels, and their corresponding microscopic images are shown in Figure 2, Figure 3 and Figure 4.

Figure 2 shows four-color immunohistochemistry staining on colorectal carcinoma. Tumor cells are stained with yellow-HRP, with pan-cytokeratin (AE1/AE3) showing a moderately to well-differentiated adenocarcinoma. Staining for Ki67 using green-HRP shows a high Ki67 index, indicating aggressive growth potential. The stained nuclei show a basal orientation in some areas of the tumor and a loss of polarity in other areas. There is a moderate background of inflammatory cells in the connective tissue between areas of carcinoma. Macrophages (CD163) are stained brown with DAB, and B-lymphocytes (CD20) are stained with blue-HRP. Box A in Figure 2 shows an area of the tumor containing a mixture of both macrophages and lymphocytes. The larger Box A shows this same area at a higher magnification.

Figure 3 depicts another moderately differentiated carcinoma showing adenocarcinoma morphology. In this example, the carcinoma cells are stained with pan-cytokeratin (AE1/AE3) using red-AP chromogen. Ki67 is stained using green-HRP and shows a moderate to high Ki67 index, indicating high growth potential (Box C). Nuclei also lost their polarization. Compared to the colorectal carcinoma in Figure 2, this carcinoma displays a substantially greater number of inflammatory cells. Macrophages (CD163) are stained brown with DAB and B-lymphocytes (CD20) are stained blue with blue-HRP. Furthermore, in this area of the tumor there appears to be some separation between the B-cells (Box A) and the macrophages (Box B). The larger boxes, A, B, and C, show the same areas at a higher magnification.

Figure 4 depicts an example of another colorectal carcinoma. The staining with red-AP for pan-cytokeratin (AE1/AE3) shows a moderately to well-differentiated adenocarcinoma. The tubular structures are widely dispersed throughout an extensive stroma (Box A). The Ki67 labeled with green-HRP shows a lower Ki67 index compared to the two previous examples of colorectal carcinoma (Figure 2 and Figure 3). There is a moderate scattering of inflammatory cells composed of macrophages (CD68, brown) and B-lymphocytes (CD79b, blue). Occasional tertiary lymphoid structures are observed and stained positive for B-lymphocytes (Box B). The larger boxes, A and B, show the same areas at a higher magnification.

## 4. Discussion

A thorough understanding of the various technologies strengths, weaknesses, and limitations is crucial in answering specific questions regarding tumor heterogeneity and the microenvironment. No single technology can provide all the answers to all the questions. Several unique technologies have been introduced to address some of these questions [14,15,16]. Morrison et al. [15] introduced the concept of invisible chromogens that have narrowly defined absorbance bands in the ultraviolet or near-infrared spectra, making these chromogens invisible within the visible spectrum. By selecting appropriate filters, the chromogens and conventional stains could be separately imaged. Other investigations have shown the development of new chromogens based on tyramine chemistry with narrow absorbance bands, making them ideal candidates for spectral imaging [16].

Here, we present a new technology based on classical IHC chromogenic methods that can be easily incorporated into most pathology laboratories without modification of existing equipment or practices. This method is based on various chromogenic substrates that provide excellent color separation. Multiplex staining is achieved by sequential IHC with antibody elution performed between each sequential step. Using this approach, we have demonstrated the feasibility of identifying up to four different markers with excellent color differentiation. Furthermore, the time required to achieve these results is within a few hours, thus making this method feasible within the typical laboratory’s daily workflow.

Depending on the specific antibody panel chosen for multiplex staining, the complexity of the information obtained can be overwhelming such that mere visual interpretation by the microscopist may be insufficient to extract all relevant information and interpret all expression patterns. Such situations can certainly benefit from computer-aided image analysis using suitable algorithms [17,18]. In such situations, spectrally distinct chromogens are critical for accurate results. In this study, we have shown the feasibility of a new method for creating multiplex IHC stains that takes advantage of existing methods and extends those methods by the introduction of a new set of chromogenic substrates. The implementation of spatial technologies is increasingly important to identify and locate specific cell types and patterns in the context of the tumor microenvironment, and this may well lead to new insights into previously unrecognized biological processes.

## 5. Conclusions

In this study, we have described a method for multiplex staining in tissue samples using standard immunohistochemical methods. This method relies on the development of a new set of chromogens that can be used individually or in combination with existing chromogens to generate multiplex stains. When used in multiplex stains, these chromogens show fine cellular detail, display low background, and retain their original colors. Chromogens exhibiting sufficient color separation from each other can be combined in such a way that simple visual interpretation of the resulting images is possible. Using this method, we have successfully demonstrated four-color immunohistochemistry. Depending on the types of staining patterns generated, visual analysis without the assistance of computer-aided image analysis may be possible.

## Figures and Tables

**Table 1:** Antibody list for multiplex staining.

Antibody	Species	Clone	Part No.
CD3 (epsilon chain)	Rabbit	SP7	RMAB005
CD4	Rabbit	EP204	RMAB053
CD5	Rabbit	SP19	RMAB11
CD8	Mouse	144B	Mob117
CD20	Mouse	L26	Mob004
CD68	Mouse	PGM1	Mob094
CD79a	Mouse	HM57	Mob118
CD163	Mouse	10D6	Mob480
Ki67	Rabbit	SP6	RMAB004
PD-1 (CD279)	Mouse	EH33	Mob573
PD-L1 (CD274)	Mouse	405-9A11	Mob572
Cytokeratin HMW	Mouse	34BE12	Mob059
Pan-Cytokeratin	Mouse	AE1/AE3	Mob190

**Table 2:** Reagent list.

Name	Part No.
Tissue Glue	K096
Wash Buffer	K005
UnoVue Anti-mouse HRP	MUHRP-100
UnoVue Anti-rabbit HRP	RUHRP-100
UnoVue Anti-mouse AP	MUAP-100
UnoVue Anti-rabbit AP	RUAP-100
Antigen Retrieval pH 9.0	K043
Primary Antibody Diluent	K004

**Table 3:** List of chromogens.

Chromogen	Part Number	Enzyme System
PermaRed-HRP	K075	Peroxidase
PermaBlue-HRP	K063	Peroxidase
PermaGreen-HRP	K074	Peroxidase
PermaYellow-HRP	K060	Peroxidase
PermaBlack-HRP	K062	Peroxidase
Stable DAB	K047	Peroxidase
PermaRed-AP	K049	Alkaline Phosphatase

**Table 4:** Effects of the elution buffer on chromogens.

Chromogen	Effect of the Elution Buffer On Chromogen Staining
PermaRed-HRP	Slight loss of staining
PermaBlue-HRP	No effect
PermaGreen HRP	Moderate loss of staining
PermaYellow-HRP	No effect
PermaBlack-HRP	Slight loss of staining
Stable DAB	No effect
PermaRed-AP	No effect

**Table 5:** Optimal panels for four-color immunohistochemistry.

Sequence	Primary Antibody	Chromogen	Tissue	Image
1	Pan-cytokeratin (AE1/AE3)	Yellow-HRP		
2	Macrophage (CD163)	DAB	Colorectal	Figure 2
3	B-cell (CD20)	Blue-HRP	Carcinoma	
4	Proliferation marker (Ki67)	Green-HRP		
1	Pan-cytokeratin (AE1/AE3)	AP-Red		
2	Macrophage (CD163)	DAB	Colorectal	Figure 3
3	B-cell (CD20)	Blue-HRP	Carcinoma	
4	Proliferation marker (Ki67)	Green-HRP		
1	Pan-cytokeratin (AE1/AE3)	AP-Red		
2	Macrophage (CD68)	DAB	Colorectal	Figure 4
3	B-cell (CD79a)	Blue-HRP	Carcinoma	
4	Proliferation marker (Ki67)	Green-HRP		

## Data Availability

The original contributions presented in the study are included in the article. Further inquiries can be directed to the corresponding author.
